# Exploring the Relationship Needs of Service Users During Crisis Interventions: A Qualitative Study

**DOI:** 10.1007/s10597-024-01372-6

**Published:** 2024-10-29

**Authors:** Larissa Steimle, Sebastian von Peter, Fabian Frank

**Affiliations:** 1 Brandenburg Medical School - Theodor Fontane, Fehrbelliner Str. 38, 16816 Neuruppin, Germany; 2 Frankfurt University of Applied Sciences, Nibelungenplatz 1, 60318 Frankfurt am Main, Germany; 3 Protestant University of Applied Sciences Freiburg, Bugginger Str. 38, 79114 Freiburg im Breisgau, Germany

**Keywords:** Relationship, Alliance, Crisis, Service user, Grounded theory, Needs

## Abstract

People in crisis sometimes seek professional support, and the relationship between service users and professionals is crucial in overcoming the crisis. To understand the relationship needs of people in crisis, 29 semi-structured interviews with service users were conducted and analyzed using a grounded-theory approach. The findings reveal that people in crisis seek a professional who is there for them, recognizes the crisis as an emergency and a solvable situation, treats them with respect, and offers individual support. Furthermore, there needs to be a general fit between professionals, the support services, and service users for a supportive relationship to be established. However, two main aspects were discovered where service users differ depending on the resources they can access during the crisis: While people with access to many resources seek a rather distant and egalitarian relationship, those with access to fewer resources prefer more intimate and hierarchical relationships with professionals.

## Background

A crisis can be defined as subjective experience threatening and overwhelming a person’s ability to deal with a specific situation using their normal problem-solving abilities, coping mechanisms, or current resources (Caplan, 1964; Nizum et al., 2020). Although situations that trigger personal crises can vary greatly, the consequences that a crisis entails for individuals are often similar. They all find themselves in an emotional state of emergency (Schleuning, 2014). Some of the people in these emergency situations turn to professional services to get help to deal with their crisis. Worldwide, a range of services exist, including acute inpatient wards and services that deal with crises in emergency departments as well as in the community (Johnson et al., 2022). This range is also seen in Germany. Some services are covered by health insurance, while others are funded by social welfare. There are major regional differences in the coverage of crisis care. In psychiatric care, support ranges from registered neurologists, psychiatrists, and psychotherapists to the psychiatric outpatient clinics of hospitals and their crisis, acute, and specialized wards. In the psychosocial field, services include community mental health services, geriatric psychiatric services, and a wide range of counselling centers for specific target groups or type of crisis. This spectrum is supplemented by the services provided by the fire brigade, rescue services, and the police (Schleuning, 2014). Therefore, crisis intervention is generally a multidisciplinary field involving social workers, psychiatrists, psychologists, nurses, and many more (Murphy et al., 2015). Although in Germany, inpatient institutional care has been the standard care modality for people experiencing a crisis, more recently, outpatient community care continues to become more important (Bramesfeld, 2023; Schwarz et al., 2020).

No matter what kind of crisis people experience or which services and professionals they turn to, a relationship between the person experiencing a crisis and the professionals working at these services must be rapidly established to avoid traumatic and coercive experiences of care (Johnson et al., 2022). It is known from psychotherapy research that the relationship between the person experiencing a crisis and the professional is a significant predictor of outcome across treatment conditions (Flückiger et al., 2018; Horvath et al., 2011). In social work, medicine, and nursing, the relationship is considered a core component of any client interaction (Eveleigh et al., 2012; Gahleitner, 2017; Hartley et al., 2020). Wampold and Flückiger (2023) compare the professional relationship to language, being present in all interactions but typically being ignored until it is disrupted or vanishes.

While there is a consensus that a professional relationship should be established as quickly as possible in a crisis situation that is handled with professional help, there are few studies on this specific relationship, especially regarding crisis support. Johnson et al. (2022) state that more research focused on the therapeutic relationship in mental health crises is needed, and Jacob (2022) argues that the quality of evidence for current crisis interventions and models for acute psychiatric care is, at best, moderate. According to Bolsinger et al. (2020), the existing research focusses mainly on scheduled, non-acute, long-term, one-on-one setting like psychotherapy (e.g. Flückiger et al., 2018; Lavik et al., 2018). Further, there is research on the professional relationship in inpatient settings such as mental health wards (e.g. McAllister et al., 2019; Taylor et al., 2024). A recent scoping review revealed that to date little is known about the relationship in the community mental health context, such as outpatient, low-threshold, voluntary settings, especially from the service user’s perspective (Steimle et al., 2024). Due to this gap in research, there are currently no findings on what kind of relationships professionals should offer, even though this relationship is essential for successfully overcoming a crisis. Therefore, this study focuses on the question of how a supportive professional relationship within a crisis situation should look like from the service user’s perspective. The aim of this study was to understand what people in crisis need regarding the professional-client relationship.

## Methods

In contrast to quantitative research, qualitative research focuses on understanding people by answering questions about the “what”, “how” or “why” of a phenomenon rather than “how many” or “how much”, which are addressed by quantitative research (McCusker & Gunaydin, 2015). As one of many qualitative approaches, grounded theory is particularly useful in exploratory research to help identify patterns and key concepts that may not be initially apparent and emerge into a theory (Corbin & Strauss, 1990). Because of the exploratory nature of the research question and the interest in understanding the needs of service users, a qualitative grounded theory study was conducted with people who experienced a crisis and turned to support services^1^.

### Research Team and Reflexivity

The research team consisted of the authors of this article as well as two multiprofessional analysis groups (each comprising 3–4 researchers). All interviews were conducted by the first author (a female social worker) as part of her doctoral thesis. She has practical experience working with people with severe mental illnesses in a long-term community mental health setting. There was no contact between the study participants and the interviewer before the study, except for one interviewee who participated in an earlier study on a different topic four years prior. The study participants were informed beforehand about the aim of the study, including the focus on the professional relationship, as well as the interviewer’s name, professional background, occupation, and the fact that the study was part of her doctoral thesis.

### Participant Selection

Using the theoretical sampling method (Corbin & Strauss, 1990), contrasting concepts were constantly added. Therefore, interviews were conducted with people who had dealt with very different types of crises (depressive crises, psychotic crises, illness of daughter etc.) in various contexts (therapy, crisis services, counselling, emergency wards) with different professionals (social workers, physicians etc.). The interviewees were of different ages and genders, had long-term or short-term relationships with professionals, and had extensive or limited experiences with the support system in Germany. To find these diverse cases, potential interviewees were recruited via outpatient crisis services and self-help organizations. Outpatient support services were contacted, and a flyer containing study details and contact information was distributed to the crisis services. Professionals then gave this flyer to people using their services. This flyer was also distributed via mailing lists of self-help organizations. The aim of contacting outpatient support services was to reach people who might be receiving support for the first time and who are therefore relatively inexperienced with the system. In contrast, people who are linked to self-help organizations are generally more experienced with the system and therefore possess far-reaching experiences with support services. People who received the flyer could then contact the first author.

### Data Collection

From July 2023 till November 2023, 29 interviews were conducted with people who received professional support to overcome a crisis. On average, the interviews lasted around 40 min. Since dealing with a crisis is a sensitive topic, in weighing the positive and negative aspects (Thunberg & Arnell, 2022), participants were given more options than just in-person interviews. The choice of whether the interview was conducted in-person, via telephone or using a video conference tool was left to the study participants. 14 interviews were conducted via video conferences, 8 by telephone and 7 in person. No participants dropped out of the study. All interviews were conducted in German. In the interviews, the following two main story prompts were used, which were tested beforehand and discussed within the research team:

1. If you think back to your last crisis, please tell me about your contact with professional services.
2. Thinking back to the contact with support service X, please tell me about the relationship between you and the professionals.

The first question was intended to create descriptions of the crisis (type of crisis, institutions contacted, etc.) and to obtain initial information about the relationship with the service and the professionals. The second question was then explicitly aimed at descriptions of the professional relationship.

Written and verbal consent was obtained from all the interviewees before the interviews began.

### Data Analysis

All interviews were recorded (only audio) and then transcribed. The analysis was based on the grounded theory approach by Corbin and Strauss (1990), using inductive analysis through simultaneously collecting and analyzing data. The data underwent open, axial, and selective coding using constant comparative analysis. In this regard, lines, sentences, and paragraph segments were reviewed to determine which codes best fit the concepts suggested by the data. Each code was constantly compared to all other codes to identify similarities, differences, and general patterns. The coding was done by different researchers within two analysis groups. Subsequently, categories gradually emerged as a result of the combined process of analyzing the data and considering what was learned from sensitizing concepts.

According to Bowen (2006), sensitizing concepts provide starting points for building analysis to produce a grounded theory. In this instance, a previously conducted scoping review (Steimle et al., 2024) served as such a starting point, for example, by deliberately contrasting outpatient and inpatient relationships. Further, sensitizing concepts give the researcher a sense of how observed instances of a phenomenon might fit within conceptual categories (Bowen, 2006), such as widely discussed relationship topics like boundary setting (e.g. O’Leary et al., 2013). A very important sensitizing concept was the demand-resource-model (Becker et al., 2004). During the analysis, it became clear that service users differ in how actively or passively they responded to the crisis. While some individuals independently organized alternative solutions to problems (e.g., lack of accessibility to professionals), others did not. Throughout the analysis, it became increasingly clear that this was related to the resources available to people in crisis. The accessibility of resources seemed to influence both the coping of the crisis and the relationship. Consequently, literature was consulted: According to the demand-resource-model (Becker et al., 2004), health is influenced by the use of internal and external resources to deal with a specific situation. Internal resources refer to mental and physical resources available to a person that generally prove to be advantageous in coping with a situation, like for example problem-solving competencies, broad knowledge, self-efficacy beliefs or good physical fitness, but also personality traits, such as being independent. External resources, include for example social resources (e.g. social support systems), professional resources (e.g. control over work), material resources (e.g. sufficient income), societal resources (e.g. education) and ecological resources (e.g. healthy food).

At successive stages, categories moved from a low level of abstraction to become major, overarching categories rooted in concrete evidence provided by the data. The coding process ended when “theoretical saturation” occurred – meaning that additional data failed to uncover new concepts about the developing theory. With the help of memos and within the analysis groups, participants reflected on and critically reviewed their own theoretical assumptions and personal backgrounds (e.g., practical experience working in a long-term community mental health setting).

The study was approved by the ethical committee of Brandenburg Medical School (E-01-20230209).

## Results

The sample consisted of 18 women and 11 men, with an average age of 47 years. The youngest person was 26 years old, and the oldest was 63. The interviewees lived all over Germany, covering 10 of the 16 federal states. Most of the interviewees mentioned having a diagnosed mental illness. For some, the diagnosis had been made years before the described crisis, while for others, the described crisis situation had led to a diagnosis. The initial contact with professionals had occurred at varying times in the past and cannot always be pinpointed to a specific day, month, or year. Some interviewees were at the end of the process of a crisis intervention during the interview. Other interviewees described a crisis that dated back many years but had since become acute repeatedly. Most of the interviewees were currently still in contact with professionals.

### Crisis Situations

The interviewees described a wide range of crisis situations that prompted them to seek professional help. The most frequently mentioned crisis situations were related to depression/depressive episodes, followed by suicidal crises. Other reasons for seeking professional support included anxiety and panic attacks, psychosis, experiences of violence/abuse, suicidal behavior of a partner, drug use, etc. Some interviewees described more than one reason for seeking help, and others recounted different crisis situations over time in which they contacted professionals. What all of the interviewees had in common was that they found themselves in at least one severe emergency situation that they were unable to cope with on their own, as this interviewee described^2^^,^^3^:

*“The symptoms were really bad and it got to the point where I actually had suicidal thoughts. […] On the way there*,* where I actually wanted to do it*,* I decided to go to my general practitioner […] and I went inside and said*,* I can’t go on*,* I’m at the end*,* I can’t get anything done*,* I can’t sleep anymore*,* we have to do something or I have to do something.” (I_24)*

In these crisis situations, the interviewees consulted a variety of services and professionals, including general practitioners, outpatient crisis services, psychiatric hospitals, ambulances, and therapists. All of these services are summarized under the keyword “support system”, which some interviewees used to refer to all of these services. The relationship varied between long-term and short-term, as well as outpatient and inpatient support. For some, it was their first contact with professional help, while others had extensive experiences within the support system.

### The Professional Relationship

In the interviews, all of the participants described supportive aspects of the professional relationship during a crisis, which are presented below. These aspects were expressed across all cases.

#### Someone Being There to Help

One important aspect of a professional relationship was the feeling that someone was there for them and was sharing the burden of the crisis.

*“I felt somewhat […] taken care of […]. I was able to hand over the responsibility*,* the burden*,* this pressure to someone.” (I_24)*

This primarily involved professionals providing timely support and demonstrating a genuine desire to help. An exceptional level of effort was particularly appreciated. Especially during a crisis, interviewees wished for professionals to be present and reliable. This included making time for the individual in crisis.

*“That I as a patient don’t have the feeling […] I have to hurry up […] because […] you’ll throw me out in five minutes. And if I haven’t said everything*,* then I’m out of luck.” (I_28)*

#### Crisis is Recognized as both an Emergency and a Solvable Situation

Another crucial factor of a supportive relationship with people in crisis was that individuals felt acknowledged and taken seriously. Professionals had to recognize crises as emergency situations and give space to the crisis, making individuals feel they could be themselves. Interviewees also appreciated when professionals could endure the crisis.

*“[…] because I felt that I was taken seriously. I didn’t have the feeling that I had to tell them a thousand things about why it was all so bad*,* that they believed me […].” (I_29)*

In addition to recognizing the crisis as an emergency situation, it was equally important that professionals gave the impression that the crisis was normal and solvable. This included conveying hope and believing in the individual’s ability to act during a crisis.

*“[…] they need to exude calmness and confidence. It’s all terrible now*,* but we’ll manage.” (I_7)*

#### People in Crisis are Treated with Respect

Interviewees emphasized that supportive relationships should consist of respectful interactions, where they felt approached with respect rather than prejudice. Part of being treated with respect included professionals being empathic, authentic, and human.

*“[…] they just treated me like a person and not like a sick child.” (I_18)*

*”I think actually just be as human as possible*,* hey do you want a cup of tea […]. First the banal things that you might do as a neighbor […].” (I_18)*

Another important aspect of being treated with respect was honesty. Bureaucracy, on the other hand, was viewed as an obstacle to being treated with respect.

*“Well*,* my observation was that the nursing staff always had a lot to write about people they didn’t even know. Yes*,* if you observed it*,* they always documented quite a lot instead of sitting down and talking to people […].” (I_26)*

#### Individual Support is Offered

Another important aspect mentioned in the interviews was the feeling of being perceived as individuals with unique needs and wishes, and that professionals listened to what the people in crisis had to say.

*“It was simply important that the person tried to understand me*,* that he didn’t deal with me systematically or in such a routine way*,* by the book […]” (I_1)*

Therefore, support had to be tailored to the specific needs of each individual.

#### Fit between Professionals, the Support Services and Service Users

In the interviews it became clear that even though all aspects of a supportive relationship were met, there was no guarantee that a supportive relationship would be established. This was due to personal and institutional aspects influencing the relationship, which were described in the interviews.

Personal factors that influenced the relationship included sympathy, expectations, role attributions, attitudes, prejudices, and gender:

*“It’s also difficult for me when men are the therapist because I don’t want to make myself look small in front of men*,* so I don’t tell them everything.” (I_27)*

Furthermore, institutional factors, such as the structure of the service, were mentioned, including aspects like sufficient time, less bureaucracy, free services, the possibility of home visits, anonymity, and the design of the rooms:

*“I think confinement creates stress and space can create relaxation and that’s why I think that when you think about the buildings*,* the rooms*,* space*,* perhaps high rooms but simply the opposite of cramped conditions.” (I_7)*

In general, there needs to be a fit between the professionals, the support service, and the service users. If, for example, the support service doesn’t provide fitting structures, service users might not receive the care they characterize as supportive. Therefore, institutional and personal aspects influence the relationship, and even if the above described aspects are met, there remains the possibility of a relationship being viewed as unsupportive.

Aside from these aspects all the interviewees had in common, there were clear differences among interviewees regarding two relationship categories: Closeness and the hierarchical level. Further adding contrasting concepts revealed that the accessibility of resources influenced these two aspects of the professional relationship.

#### Different Relationship Needs Based on Accessibility of Resources

**Fig. 1:**
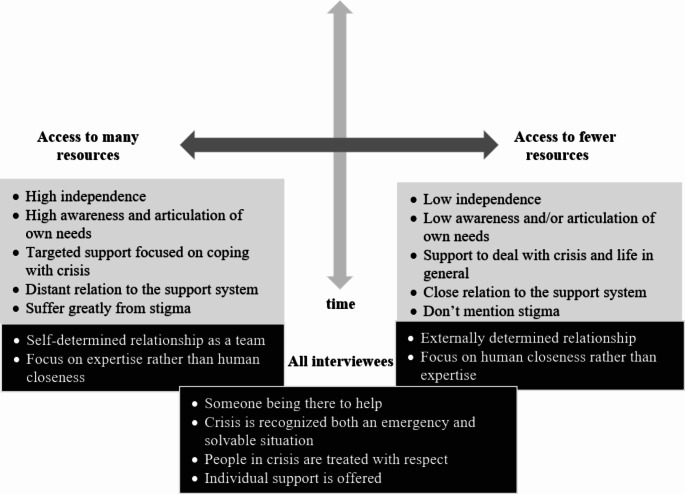
Continuum of relationship needs

The interview data showed that people with different resources had different relationship needs. On the one hand, there were interviewees who were generally able to access their own resources or had a lot of resources that could be activated within a crisis situation. On the other hand, there were interviewees with fewer resources or who were currently not accessing existing resources while dealing with the crisis. Although these two types of individuals clearly stood out in the interviews, there were certainly intermediate cases, which is why the picture of a continuum is used to describe the differences among people experiencing a crisis and accessing the support system (Fig. 1).

Interviewees were positioned on the continuum based on how they managed their last crisis. However, interviews with individuals who described more than one crisis indicated that the positioning on the continuum could change over time. Therefore, the figure also includes a temporal dimension (Fig. 1). For instance, interviewees who accessed many resources during their last crisis described earlier crisis situations where they had accessed fewer resources. The type of crisis – for example, if a person was experiencing depressive symptoms or panic attacks – did not influence resource availability. However, the data suggested that the severity of a crisis influenced the accessibility of resources and therefore the positioning on the continuum. The more severe a crisis was, the fewer resources could be accessed as this interviewee stated:

*“And when I’m depressed*,* nothing really works. I really have a switch of either zero or one*,* there’s nothing in between.” (I_13)*

##### People Who Have Access to Many Resources during Their Crisis

People on the left side of the continuum who had access to many resources generally showed a higher level of independence. Professional support is seen as complementary to one’s own coping with a crisis. They described a high awareness of their own needs during the crisis and an ability to independently organize support. Access to the support system was initiated by the individuals themselves. The search for support was targeted or tailored to the specific crisis.

*“I did not need a crisis service at first*,* so to speak*,* but rather therapeutic support […]. So*,* I sat there for a week or so and made phone calls […].” (I_29)*.

Their relationship with the support system was relatively distant, and they often referred to it as “the system” (I_29) or “doctors”/”social workers” (I_26) rather than talking about specific services or naming professionals. People who could be positioned on this side of the continuum during their crisis suffered greatly from the lack of accessibility of services and the lack of transparency of the support system. Individuals therefore often wished for a guide to help them navigate through and get access to the system. Some of the interviewees described that the lack of accessibility was worsening their situation.

*“I’m relatively open-minded and I think I can really take care of things when I’m in a good mood. But it does hurt when you leave a message on someone’s phone and they don’t call you back […]. That drags you down even more.” (I_13)*

Some individuals even considered exaggerating their symptoms to access services, as this interviewee described:

*“But they just didn’t have any room. I then had the choice to go to a closed psychiatric ward because I didn’t want to live anymore […]. But that wasn’t the case*,* but I was in a very bad place. But I couldn’t imagine myself on a closed ward. It’s always a difficult choice.” (I_29)*

Societal prejudices and stigma also played a significant role in relation to the distance to the support system. The interviewees described how the stigma of mental illnesses caused them to hesitate for a long time before seeking professional support and to conceal their diagnosis afterwards.

*“It was also simply an incredible threshold because it was socially stigmatized*,* or at least that was my impression at the time. Yes*,* psychiatry*,* only the crazy people go there.” (I_7)*

Among other things, this led to suicidal tendencies being concealed and treatment being organized via the private sphere.

*“And at the beginning I really had a big problem with running it through the health insurance company […] and then I did everything secretly*,* undercover at first.” (I_23)*

It was hoped that mental illness in general would be given greater social priority, for example by expanding support services. Another important point for some interviewees, which is closely linked to the topic of stigma, is ‘work’ as a valuable asset in society. Some interviewees feel guilty about not being able to work.

##### Professional Relationship Needs of People Who Have Access to Many Resources

Regarding the professional relationship, the people on this side of the continuum viewed themselves on the same hierarchical level as the professionals. Crises were managed as a team, but the responsibility of dealing with the crisis remained with the person in crisis.

*“Sitting there [talking to the professional] I was able to sort myself out […] and to remember things that I actually already knew […] that […] have helped me before” (I_29)*

People on this side of the continuum primarily wanted expertise from the professionals, with emotional aspects being of secondary importance. They appreciated a high level of anonymity. Not all information was shared with the professional.

*“And I haven’t even talked to my doctor about it yet*,* because I only tell him what I want to […].” (I_3)*

If the particular expertise that was expected or wished for couldn’t be or wasn’t given, the support wasn’t viewed as helpful. Professionals, for example, needed know-how on the specific type of crisis and on how to deal with it.

*“But I have to be honest*,* they were really nice. But the people were not well-trained so that they could have helped me with my serious illness. I need a therapist who knows a bit about bipolar disorder” (I_13)*

Furthermore, it was judged negatively if a professional revealed personal problems or their own vulnerability.

*“Oh yes*,* and then she called me to postpone the appointment and then told me her life story*,* which is completely unprofessional*,* that she had attempted suicide and had an abortion*,* that’s none of my business. Well*,* I found it completely unprofessional.” (I_13)*

Even if the relationship was rather distant, the ‘wavelength’ had to have been right. Both long-term and short-term relationships were viewed as helpful. Mostly, crises were dealt with in outpatient settings; however, inpatient psychiatry was sometimes used.

##### People Who Have Access to Fewer Resources

On the other side of the continuum, people with fewer resources showed a lower level of independence during a crisis. They tended to be rather unaware of their needs or were unable to or simply didn’t articulate them within the interviews. The perception of a crisis was often viewed as the responsibility of the professionals.

*“[…] and my counsellor at the time*,* she was great*,* understood very well*,* so she noticed that something was wrong.” (I_5)*

The professional support was not only focused on dealing with a crisis; it was also about providing support in coping with life in general. The interaction with the professionals was often about reassurance with daily life decisions.

*“Then I noticed a lump on his belly […] and I took him to the vet […]. Yes*,* there’s nothing more you can do […]. And then I told [PROFESSIONAL]*,* he said*,* ‘why don’t you get a second opinion? I recommend the clinic [NAME]’. […] He said ‘we had our cat [NAME] operated at clinic [NAME]’. […] [PROFESSIONAL] also helped me with this. Other psychiatrists wouldn’t even bother with that.” (I_25)*

Stigma and its influence were not a topic within the interviews. The relation to the support system was rather close and was often described as home. Professionals navigated through the system.

*“[…] I was part of the inventory of the clinic*,* that was a nice experience […].” (I_5)*

##### Professional Relationship Needs of People Who Have Access to Fewer Resources

Relationships with professionals during the crisis tended to be more intimate and were explicitly characterized as friendships. Physical contact was increasingly important. Anonymity was not mentioned as an important factor. When forming relationships with professionals, human closeness was crucial, while professional expertise played a subordinate role.

*“[PROFESSIONAL] is very okay. So*,* we also hug each other and he’s very nice and stuff and helps me and once gave me a six-pack of beer for my birthday in 2017*,* shortly after we started working together*,* because he knew I drink beer.” (I_25)*

*“Yes*,* and we also talk about sexuality and I can talk to him like a friend*,* he smokes with me in his office. Most people don’t do that.” (I_25)*.

People with fewer resources were more likely to have long-term relationships, although short-term relationships were also considered helpful in some cases. Many of the interviewees received long-term inpatient and outpatient care.

## Discussion

Other studies in the context of professional relationships have focused on aspects and characteristics of supportive relationships in specific settings, such as acute mental health units (e.g. Moreno-Poyato et al., 2023). This study, however, adopted a service-independent approach, aiming to understand the relationship needs of people who find themselves in crisis.

In this study, it was found that people experiencing an acute crisis seek professionals who are there for them, recognize the crisis both as an emergency and a solvable situation, treat them with respect, and offer individual support. Additionally, a general fit between professionals, support services, and service users is crucial for establishing a supportive relationship. However, two main aspects were identified where service users differ depending on the resources they are able to access during a crisis: While people who have access to many resources during a crisis prefer a more distant and egalitarian relationship, those with access to fewer resources often favor a close and more intimate relationship that is rather hierarchical (Fig. 1).

### Relationship Needs of all Service Users

The aspects identified as important for all individuals in crisis align with current relationship literature. There is evidence on the importance of professionals being there for them (e.g., making time, offering timely support, being reliable (Giménez-Díez et al., 2020; Magill et al., 2022; Nizum et al., 2020), taking the crisis seriously/conveying hope (e.g., offering hope, not being phased by it all (Hopkins & Niemiec, 2007; Wampold & Flückiger, 2023), showing respect (e.g., showing empathy, respect, not being judgmental (Hopkins & Niemiec, 2007; Ljungberg et al., 2016; Wampold & Flückiger, 2023), and offering individual support (e.g., being treated as an individual, being listened to (Hopkins & Niemiec, 2007; Magill et al., 2022). Furthermore, there is also considerable evidence regarding the fit between professionals, support services, and service users, for example regarding role-expectations (Abplanalp et al., 2020). Part of the definition of alliance explicitly mentions “goal consensus” as an important part of the relationship (Schnur & Montgomery, 2010). Moreover, personal factors (e.g., capacities to form an alliance, attachment histories, attachment styles, motivation, needs for affiliation, and self-stigmatization (Dubreucq et al., 2021; Wampold & Flückiger, 2023) and institutional factors (Bolsinger et al., 2020), such as accessibility of the service (Magill et al., 2022), are known to influence the relationship.

### People with Different Resources

Furthermore, what was found in this study is that people with varying resources find themselves within the support system. These individuals can be situated on a continuum, with those who are currently able to access their own resources or have a lot of resources that can be activated within a crisis situation at one end, and those that are currently not able to activate existing resources or generally have fewer resources to fall back on being on the other. That people have access to a different amount of resources within a crisis situation surely isn’t a new discovery as the International Classification of Functioning, Disability and Health (ICF) – a framework provided by the World Health Organization for measuring health, functioning, and disability at both individual and population levels – is based on the idea that different people have different abilities (World Health Organization, 2024). Even though different terms are used – such as resources, capacities, or abilities – people vary in regard to what they bring to the table to deal with a crisis. This further influences people’s response to a crisis, as described in the literature (Newbigging et al., 2020) and is even part of the crisis definition itself (Caplan, 1964; Nizum et al., 2020).

It should be emphasized once again that people were positioned on the continuum based on the resources available during the crisis, not according to how many resources they have in general (e.g., after or before the crisis). As already described, there are far-reaching indications that the same person can draw on many resources in one crisis situation and fewer resources in the next crisis situation, or vice versa. Therefore, even though there are hints within the data that resources can be gained as a result of a crisis, existing literature makes it clear that most of these resources were already built up before the crisis. Studies show that the opportunity to build up resources before a crisis is influenced by various factors, including socio-economic status, race, and gender (Williams et al., 2010). While this study did not focus on these factors, no evidence was found on their influence among study participants. Furthermore, within our data we found no hints that the type of crisis influences the localization of people on the continuum, even though existing literature suggests otherwise. Pounds (2017), for example, states that “deficits in the social repertoire of individuals with schizophrenia have implications for the growth and development of the nurse-patient relationship” (Pounds, 2017, p. 193). Browne et al. (2018), for example, found that more severe positive and less severe negative symptoms were significantly and uniquely associated with a better therapeutic alliance with people experiencing their first psychosis. The reason for this could be that with respect to the needs of the relationship, the severity of the crisis is probably more important than the type of crisis. Nevertheless, we cannot rule out that there is an influence of the type of crisis. Further research is needed to explore these links and influential factors related to the relationship.

### Relationship Needs of People with Different Resources

Even though it is known that people in crisis have access to a different amount of resources, regarding the existing literature on professional relationship-building during crisis interventions, different needs from different people are rarely mentioned (e.g. Giménez-Díez et al., 2020; Hopkins & Niemiec, 2007). What this study therefore adds is the idea that different people with different resources, abilities, and perspectives have different relationship needs. In this case, regarding two dimensions: closeness and the hierarchy between service users and professionals.

The interplay between closeness and distance is part of the discussion about professional relationships in all of the disciplines involved in crisis interventions, including social work, psychotherapy, medicine, and nursing. This phenomenon is discussed under keywords like “boundary setting” (e.g. O’Leary et al., 2013) or “dual relationships” (e.g. Vesentini et al., 2021), meaning that the professional has two or more roles in their relationship with the person with whom they engage in the course of their professional practice (Crowden, 2008). In most of the publications a continuum “between the ‘professional, objective expert’ and the ‘helpful friend’” (Green et al., 2006) is described. The experienced practitioner is moving along on the continuum between each stance to meet the demands of the particular context (Green et al., 2006). However, many publications highlight the dangers of intimacy and the importance of setting boundaries (Gardner et al., 2015). Further, even though in literature it is mentioned, that the professionals should aim for an egalitarian relationship (Kam, 2021), it is acknowledged that power imbalances always exist (O’Leary et al., 2013). According to this study, there needs to be a more open discussion about closeness and hierarchy in relationships, with regard to the current resource situation of people in crisis.

### People with Access to Many Resources and the Support System

In addition to these aspects relating to the relationship with professionals, what was also found is that especially people with access to many resources expressed problems in relation to the support system. People who have many resources to fall back on appear to be overwhelmed by the confusion regarding responsibilities and the lack of accessibility, while people with access to fewer resources are guided through the system by professionals. This is particularly worrying because of the harmful short-term effects and potential long-lasting effects that a crisis can entail if timely and effective interventions are missing (Nizum et al., 2020). It can be hypothesized and some interviewees even describe that because of this problem, people can lose (some of) their resources. There is therefore a need for greater transparency regarding which services are available to which groups. Schleuning (2014) already criticizes the support system in Germany as being very complex, making it difficult for people in crisis to know who is responsible for which type of crisis. Therefore, according to Gaebel (2020) care coordination is needed. Further it is criticized that not enough support exists (Schleuning, 2014).

Another reason endangering immediate crisis support mentioned in the interviews is stigma. Within the interviews it is described that perceived stigma leads to barriers to help-seeking. This aligns with current literature (e.g. Knaak et al., 2017; Schnyder et al., 2017). Outpatient crisis services in particular can offer structural solutions here (e.g. anonymous, free crisis interventions) to counteract this barrier to seeking help. However, this requires more widespread availability of outpatient crisis services and better advertising of these services. Of course, a more comprehensive initiative is needed to address stigma as a whole.

## Limitations

This is a qualitative study assessing the subjective experience of service users. Even though there is evidence that the positive influence of a professional relationship is fundamentally determined by the subjective perception of the person in crisis (Fitzpatrick et al., 2005), we didn’t assess whether the relationship led to a good outcome in regard to overcoming a crisis.

Furthermore, the contact with professionals during the crisis occurred at different times, which may have led to differing levels of recollection. The crisis situation itself might have further influenced the ability of service users to recall information at a time of acute distress. Further, there surely are some methodological limitations. Within this study interviews were conducted via telephone, video conference tools and in-person. Even though positive and negative aspects were carefully weighed, this may have influenced the interview data, like missing gestures or facial expressions. In addition, our access to the field via crisis services and self-help groups may have targeted only certain people, while other groups of people may not have been reached.

Overall, however, we are convinced that our data meet the standards of qualitative research, despite the limitations.

## Conclusion

This study highlights that people in crisis who seek professional support have overlapping needs regarding the relationship. Apart from these relationship factors that are viewed as supportive by all service users, there are two aspects where people differentiate with respect to their resources: closeness and hierarchy. This diversity of individuals concerning their relationship needs is, to date, hardly discussed in the literature. To meet the needs of service users, discussion and reflection among the professionals involved in crisis intervention are necessary to address people’s needs for either a closer, hierarchical or a more distanced, egalitarian relationship.

## Footnotes

1 The term “support services” explicitly refers to all types of facilities (outpatient, inpatient, long-term, short-term).

2 As all of the interviews were conducted in German, the original quotes were translated by the authors.

3 To maintain readability, for the purpose of this article the interviews were smoothed out (e.g., filler words removed, pause indicators shortened).
